# Supplementary figures and images for: A novel approach to interrogating the effects of chemical warfare agent exposure using organ-on-a-chip technology and multiomic analysis
Source: PLoS One. 2023 Feb 13;18(2):e0280883. doi: 10.1371/journal.pone.0280883 (PMC9925079; doi:10.1371/journal.pone.0280883)

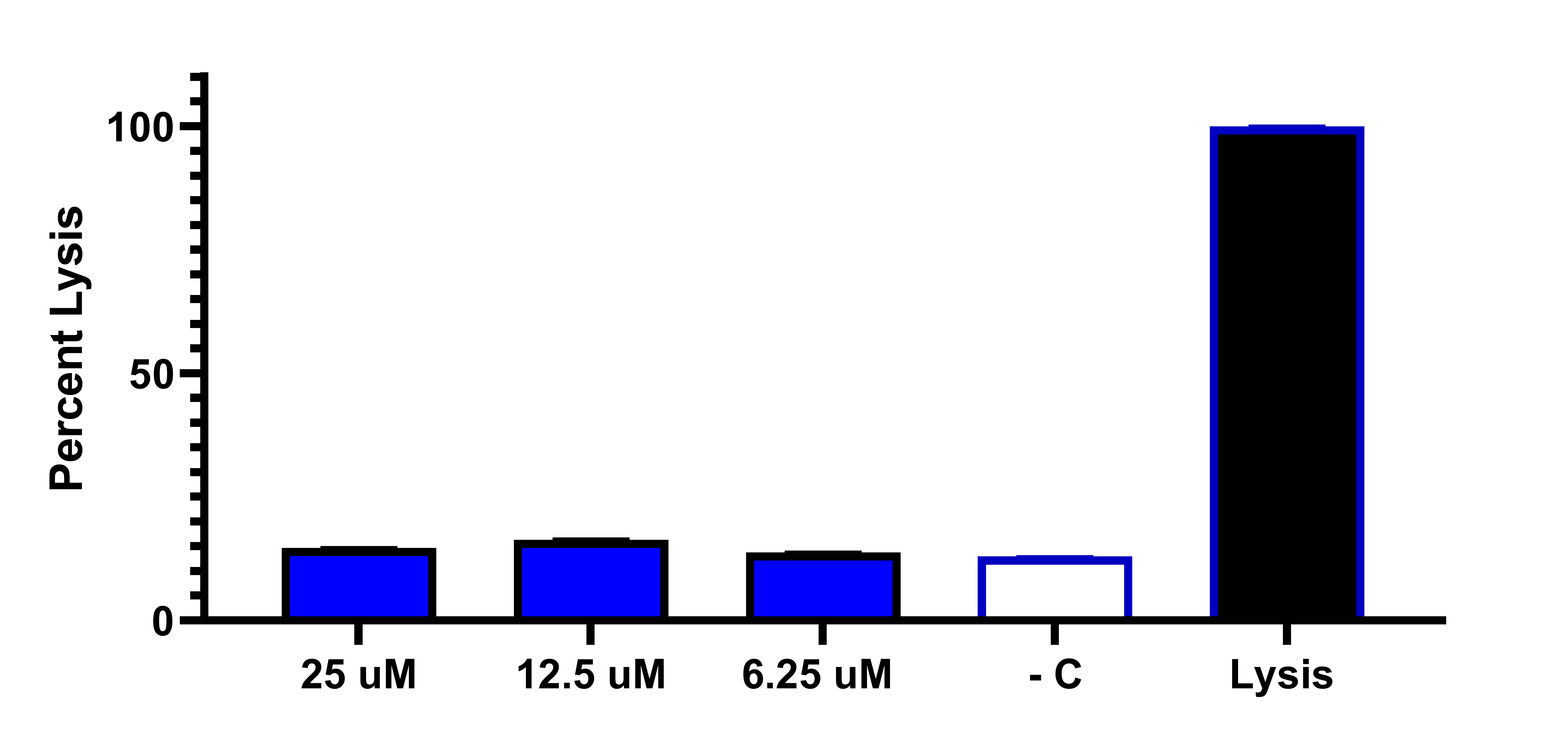

Supplement: S1 Fig — (TIF) [file pone.0280883.s001.tif]

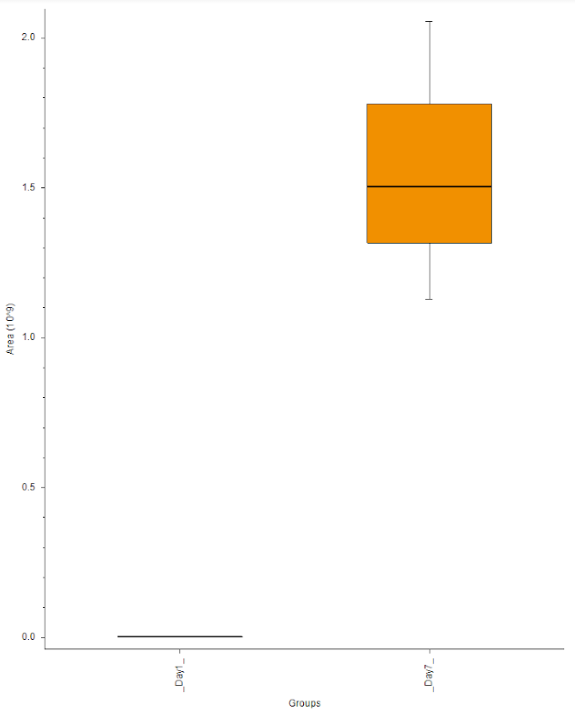

Supplement: S2 Fig — (TIF) [file pone.0280883.s002.tif]
