# Supplementary material for: A novel approach to interrogating the effects of chemical warfare agent exposure using organ-on-a-chip technology and multiomic analysis
Source: PLoS One. 2023 Feb 13;18(2):e0280883. doi: 10.1371/journal.pone.0280883 (PMC9925079; doi:10.1371/journal.pone.0280883)
Supplement: S1 Appendix — (DOCX) [file pone.0280883.s007.docx]

**Appendix A**

The following medium preparations were used in this study:

Base LSEC Culture Medium (500 mL)

CSC basal medium (Cell Systems) - 485 mL

Culture-boost (Cell Systems) - 10 mL

Pen / strep (Sigma Aldrich) - 5 mL

Complete LSEC Culture Medium (50 mL)

Base LSEC Culture Medium- 45 mL

FBS (Sigma Aldrich)- 5 mL

Base Hepatocyte Seeding Medium (500 mL)

WEM + (with phenol red) (Sigma Aldrich) - 490 mL

Pen / strep - 5 mL

L-GlutaMax (Thermo Fisher) - 5 mL

Complete Hepatocyte Seeding Medium (200 mL)

Base Hepatocyte Seeding Medium - 187.78 mL

ITS+ premix (Corning)- 2 mL

Ascorbic Acid - 200 µL

Dexamethasone (10 mM) - 20 µL

FBS - 10 mL

Base Hepatocyte Maintenance Medium (500 mL)

WEM - (without phenol red) (Sigma Aldrich) - 490 mL

Pen / strep - 5 mL

L-GlutaMax - 5 mL

Complete Haptocyte Maintenance Medium (50 mL)

Base Hepatocyte Maintenance Medium - 49.445 mL

ITS+ premix - 500 µL

Ascorbic acid - 50 µL

Dexamethasone (1mM) - 5 µL

Hepatocyte Overlay Medium

Complete Hepatocyte Maintenance Medium - 19.5 mL

Matrigel - 0.5 mL

Non-Parenchymal Cell (NPC) Seeding Medium (50 mL)

Complete Hepatocyte Maintenance Medium omitting dexamethasone - 22.5 mL

Base LSEC Culture Medium - 22.5 mL

FBS - 5 mL

NPC Maintenance Medium (50 mL)

Complete Hepatocyte Maintenance Medium omitting dexamethasone - 24.5 mL

Base LSEC Culture Medium - 24.5 mL

FBS - 1 mL
