# Supplementary material for: A novel approach to interrogating the effects of chemical warfare agent exposure using organ-on-a-chip technology and multiomic analysis
Source: PLoS One. 2023 Feb 13;18(2):e0280883. doi: 10.1371/journal.pone.0280883 (PMC9925079; doi:10.1371/journal.pone.0280883)
Supplement: Attachment — Submitted filename: PONE-D-22-07224 Rebuttal Letter.docx [file pone.0280883.s008.docx]

**PLOS ONE (PONE-D-22-07224)**

**Response to Reviewers**

**Authors:** Goralski TDP, Jenkins CC, Angelini DJ, Horsmon JR, Dhummakupt ES, Rizzo GM, Simmons BL, Liem AT, Roth PA, Sekowski JW, Glover KP

**Title:** A Novel Approach to Interrogating the Effects of Chemical Warfare Agent Exposure Using Organ-on-a-Chip Technology and Multiomic Analysis

**ID #:** PONE-D-22-07224

**Comments from the Editor:**

**1. Please ensure that your manuscript meets PLOS ONE's style requirements, including those for file naming.**

All style requirements have been met.

**2. Please ensure you have included in your Methods section full information regarding the origin (supplier or manufacturer) of all materials used in this study. This includes in particular the origin of the VX chemical warfare agent.**

Full information regarding the origin of all materials used in this study, including the supplier and manufacturer, have been included in the manuscript. The origin of the VX chemical warfare agent has been included, as well as the purity of the sample.

**3. Please remove any funding-related text from the manuscript and let us know how you would like to update your Funding Statement.**

Funding information was removed from the Acknowledgements section of the manuscript. The information provided in the review is correct.

**4. In your Data Availability statement, you have not specified where the minimal data set underlying the results described in your manuscript can be found.**

All data sets have been uploaded to repositories, and we are awaiting accession numbers. The transcriptomics data were uploaded to Gene Expression Omibus through NCBI, the proteomics data were uploaded to the PRIDE Proteomics Identification Database, and the metabolomics data were uploaded to the Metabolights Database. We will provide you with the accession numbers as soon as possible.

**5. We note that you have stated that you will provide repository information for your data at acceptance. Should your manuscript be accepted for publication, we will hold it until you provide the relevant accession numbers or DOIs necessary to access your data. If you wish to make changes to your Data Availability statement, please describe these changes in your cover letter and we will update your Data Availability statement to reflect the information you provide.**

The data availability statement will remain the same. See above comment.

**6. We note that you have included the phrase “data not shown” in your manuscript. Unfortunately, this does not meet our data sharing requirements.**

Figure S1 was added to the manuscript, which shows the LDH cytotoxicity data described.

**Reviewer(s)' Comments to Author:

Reviewer: 1

Comments to the Author**

**1. The use of jargon, combined with problematic sentence structure and poor grammar, made several parts of the results and discussion/conclusions sections difficult to follow. Further, the organization of these sections could be improved. Notably, what the authors consider "method validation" and what they consider new findings are not clearly differentiated.**

The manuscript was revised to tone down, or better explain the use of jargon, and correct all problematic sentence structure and poor grammar. Method validation and novel findings were defined in the discussion section. The manuscript states that through validation of this novel methodology, we were able to identify both previously implicated and unique pathways associated with VX exposure.

**Reviewer: 2**

**Comments to the Author**

**1. Methods, Hepatocyte Matrigel Overlay (line 432). Authors describe the use of Matrigel for the culturing of their organ-on-a-chip system, but don’t discuss the potential interference in the metabolomics from this highly complex media or if the Matrigel was effectively washed/removed prior to extraction.**

The chips were washed 3X with DPBS before the tissues were removed for downstream processing and analysis. All chips, including those that were untreated, received the same lot of Matrigel purchased from Corning (9119020), so any effects on the metabolomics would be equally observed in both control and treated chips. Therefore, these potential effects would not stand out as off target dysregulations in the VX exposed chips.

**2. Discussion (line 316). Authors compare the organ-on-a-chip results as comparable to in vivo results but fail to show sufficient evidence that the low extraction had sufficient metabolic information to derive physiological information relative to the toxicity treatment. More direct comparisons are needed to determine that this system and low extraction are sufficient.**

We noted multiple times throughout the manuscript that some of the results obtained from the chip exposures match the dysregulations observed *in vivo*, with references to previous studies using animal models for VX exposures and downstream omics analysis. We believe that this is validation of our low extraction method.

**3. Discussion (line 351). Authors again discuss how the results are comparable to the in vivo results but do not show this sufficiently with the interference of the plasticizers.**

Additional comments were made to the Discussion section to address the potential for plasticizer interference. “Given that the plasticizers are a likely byproduct of the organ chip systems, it is improbable that these off target effects would be observed in multiomics data sets obtained from similar exposures in vivo. However, in our assessment, effects that would implicate the specific plasticizers identified by omics analysis were not reflected in our data.

**4. Identifications referenced in Figure 3 are hard to see.**

Figure 3 identifications were edited for clarity.

**Reviewer: 3**

**Major Comments to the Author**

**1. Why were the RP negative, HILIC positive, and HILIC negative results not presented or discussed?**

HILIC Positive sPLS-DA data was included in the manuscript. All acquisitions are included as supplemental data files.

**2. Method details on the partial least squares and the linear regression analysis need to be included. Include an explanation of the color scale in the heat maps.**

The sPLS-DA methodology is built into the search engine utilized for the metabolomics experiments. Compound Discoverer’s sPLS-DA algorithem is based on the paper entitled Sparse PLS discriminant analysis: biologically relevant feature selection and graphical displays for multiclass problems. This reference has been added to the manuscript. The color mapping of the heatmaps has been declared in the figure legend.

**3. The metabolomics results description was confusing in that a “top 10” was mentioned, but then only 3 were said to be dysregulated. Please elaborate.**

We have clarified this by stating that out of the ten discriminating compounds, only three were found to be significantly dysregulated.

**4. The phrase “sPLS-DA was performed incorporating time of day” is confusing because time of day is not mentioned previously. Do the authors mean days post-exposure?**

This was revised in the manuscript.

**5. The results/discussion for the plasticizer contamination was unclear. Were they increased in the Day 7 samples as one might expect?**

This was revised in the results section, and the octyl isocyanate boxplot was added to the supplemental.

**6. Regarding days post-exposure, the authors speculated that “One possible answer is that basal level activity within the tissues could mature the longer they are in the chips.” Can’t the untreated controls be used to normalize for such age-associated changes?**

**7. The authors should double check the p-value thresholds. Figure 8, for example, is described as both p=0.005 (caption) and p<0.05 (text).**

The manuscript was revised to reflect that the p-value was 0.005.

**8. My understanding of S-plots was that the extremes were interesting (upper right and lower left). The 10 points highlighted in figure 2 seem to be randomly scattered in the S-plot. Therefore, I do not understand what I am supposed to conclude from the plot and how it relates to the highlighted points.**

The sPLS-DA algorithem in Compound discoverer places additional factors into its identification of sPLS-DA compounds. It takes into account feature presence across all sample groups and the extremes are some features that appear in few or none of the samples in a sample group thus skewing the discrimination capabilities.

**Minor Comments to Author**

**1. The method section on VX solution preparation cuts off mid sentence •**

The methods section on VX solution preparation was revised.

**2. The caption in figure 4 has B and C backwards.**

Figure 4 caption was revised

**3. Page 8: incidents or incidence?**

Changed to incidence.

**4. Page 4: compliments or complements?**

Changed to complements.

**5. Page 12: “While we observed high p-values”…should this be low p values?**

Changed to low p-values.
