# Supplementary material for: A novel approach to interrogating the effects of chemical warfare agent exposure using organ-on-a-chip technology and multiomic analysis
Source: PLoS One. 2023 Feb 13;18(2):e0280883. doi: 10.1371/journal.pone.0280883 (PMC9925079; doi:10.1371/journal.pone.0280883)
Supplement: Attachment — Submitted filename: PONE-D-22-07224 Rebuttal Letter.docx [file pone.0280883.s009.docx]

**PLOS ONE (PONE-D-22-07224)**

**Response to Reviewers**

**Authors:** Goralski TDP, Jenkins CC, Angelini DJ, Horsmon JR, Dhummakupt ES, Rizzo GM, Simmons BL, Liem AT, Roth PA, Karavis MA, Hill JM, Sekowski JW, Glover KP

**Title:** A Novel Approach to Interrogating the Effects of Chemical Warfare Agent Exposure Using Organ-on-a-Chip Technology and Multiomic Analysis

**ID #:** PONE-D-22-07224

**Reviewer(s)' Comments to Author:

Reviewer: 1

Comments to the Author**

**1.** I agree with the authors that organ-on-a-chip technology has tremendous potential in this space. I still have concerns regarding the discussion of method validation in this paper. It seems the claim that the organ-on-a-chip design is valid and accurate for assessing the effects of VX on liver tissue is based on general similarity of some results with generally established effects of VX. For a proof-of-concept study, I would expect some outlining of explicit parameters, as established a priori, to assess method performance relative to more established cell-line and in vivo methods. While similarity of some results with expected results is good, it isunclear if unexpected results are informative - especially given the high variation observed for the untreated chips and findings regarding carcinogenic plastic contamination. While I do not think the fact that these questions were not answered by the study is in and of itself a problem, I do think the repeated language that the study showed the method is valid and accurate and reveals effects of VX not previously established by other methodologies is problematic. Further, the authors seem to frame this method as a substitute or surrogate for in vivo studies, which is not the intent of organ-on-a-chip technology. It is typically viewed as a bridge between cell lines and in vivo studies.
In sum, the study is promising and innovative, and possible issues are admittedly mentioned, but the authors have used strong language in their conclusions that should be tempered or nuanced at this stage.

The manuscript was revised to remove and amend all language that would suggest this study was a validation of the method, as well as language which would indicate the method was fully accurate, or proof of concept.

**Reviewer: 2**

**No Comments to the Author**

**Reviewer: 3**

**No Comments to the Author**
